# Corrigendum

**DOI:** 10.1111/os.13387

**Published:** 2022-07-03

In [1], the author noticed that the first affiliation was written incorrectly. The correct affiliation should be as follows:

^1^Nanfang Hospital, Southern Medical University, Guangzhou, China.

The authors apologize for the error and any inconvenience it may have caused.
